# Challenges and considerations of DNA methylation markers in peripheral blood mononuclear cells for early screening of lung adenocarcinoma

**DOI:** 10.1097/JS9.0000000000002245

**Published:** 2025-01-29

**Authors:** Rong Fu, Huijie Li, Xiurong Li

**Affiliations:** a Shandong University of Traditional Chinese Medicine First Clinical Medical College, Jinan, China; b Shandong University of Traditional Chinese Medicine Affiliated Hospital, Jinan, China

*Dear Editor,*

We recently read with interest the article “Multi-site DNA Methylation Alterations of Peripheral Blood Mononuclear Cells Serve as Novel Biomarkers for the Diagnosis of AIS/stage I Lung Adenocarcinoma: A Multi-center Cohort Study” by Li *et al*^[1]^, published in the *International Journal of Surgery*. A multicenter cohort study was conducted to evaluate the association between PBMC methylation and lung adenocarcinoma. Among them, 1415 people from nine medical centers were included in the analysis, and the results showed that the methylation differential sites of PBMC could be used as reliable markers for the early diagnosis of lung adenocarcinoma, and the LDP score was successfully developed to quantify the risk of lung adenocarcinoma.

Early-stage lung adenocarcinoma includes adenocarcinoma in situ and stage I, which is localized. The 5-year survival rate after radical surgery is more than 95%, but it is only about 10% for stage IIIA-IV patients^[2]^. Therefore, early detection and radical surgery are the key to improve the 5-year survival rate. In patients with CEA negative and unclear CT diagnosis of pulmonary nodules and GGN (6–20 mm), the timing of surgery is often difficult to determine, and the surgical strategy is also controversial. In this study, LDP score showed an AUC performance of 0.928 and 0.854 for the detection of AIS and MIA, respectively, and was specific for lung cancer, with higher predictive power than existing blood-based methylation markers. This can provide important surgical indications for surgeons, and provide a possible diagnostic approach for patients whose high-risk signs in CT images are not significant and follow-up time is long and it is difficult to decide whether to perform surgery.

As with any new technology, the cost of infrastructure for PBMC extraction and methylation testing poses a major barrier to widespread treatment, and no study has evaluated the cost-effectiveness of using PBMC methylation testing as an early diagnosis of LUAD. Since the treatment of LUAD is relatively expensive for a subset of patients, regardless of disease stage, this is particularly relevant for the scalability of methylation testing. Currently, few large-scale studies have evaluated its applicability in early screening of lung adenocarcinoma. The sensitivity and specificity of methylation detection in PBMC for monitoring and diagnosis have been validated by multicenter prospective trials. However, further development of its clinical application depends on making it a cost-effective and scalable option, which depends on ensuring that its accuracy and reliability match or replace current options.

In addition, differences in DNA methylation may be influenced by the cellular composition of PBMC^[3,4]^. Current studies should consider the effect of inclusion of immune cell subpopulations on methylation function. The proportion of B lymphocytes in early lung adenocarcinoma begins to increase, but the B cells in PBMC are less than 2%, which may lead to the omission of characteristic information of B cell subsets. The distribution of immune cells is different in different disease states and ages, and the methylation characteristics are also heterogeneous. It is necessary to exclude false positive sites caused by changes in cell proportion in PBMC after correction of cell components by the EpiDISH procedure, which may significantly improve the detection rate of methylation markers in blood. Therefore, further efforts should be made in these areas.

Second, the recurrence of lung adenocarcinoma after radical surgery is the focus of surgeons, and the 2-year recurrence rate of stage I-IIIA lung adenocarcinoma is about 20%-50%^[5]^. The best prognosis was observed in the adherent type of adenocarcinoma, and the solid subtype was proved to be a poor prognostic factor in the treatment of early lung cancer with sublobar resection. At present, it is difficult to accurately determine the risk subtypes by intraoperative frozen section because of the differences in pathological techniques in different regions. Current studies of methylation detection rarely compare with histological grade, thus ignoring the problem of recurrence after diagnosis. Therefore, it is suggested that the detection of methylation sites should also be stratified in the histological classification of lung adenocarcinoma, which may help the intraoperative surgical method.

If targeted management of high-risk LUAD populations may benefit from these alterations, the detection of PBMC methylation may have great potential for early cancer screening.

## Data Availability

Not applicable.
